# Recurrent divergence-insufficiency esotropia in Machado-Joseph disease (spinocerebellar ataxia type 3)

**DOI:** 10.1016/j.ajoc.2022.101754

**Published:** 2022-11-12

**Authors:** Jeannette Y. Stallworth, Nailyn Rasool, Maanasa Indaram

**Affiliations:** a Department of Ophthalmology, University of California, San Francisco, United States; b Department of Neurology, University of California, San Francisco, United States

**Keywords:** Divergence-insufficiency esotropia, Machado-Joseph disease, Spinocerebellar ataxia

## Abstract

**Purpose:**

To describe a case of incomitant divergence insufficiency esotropia in the setting of Machado-Joseph disease (spinocerebellar ataxia type 3) that recurred completely within one week after augmented bilateral medial rectus recession.

**Observations:**

A 53-year-old female with a history of Machado-Joseph disease presented with horizontal diplopia primarily at distance consistent with divergence insufficiency esotropia. Augmented bilateral medial rectus recessions were performed which initially produced orthotropia, but recurrence of the esodeviation to the full preoperative amount occurred by post-operative week one. The patient subsequently underwent bilateral lateral rectus resections with excellent result.

**Conclusions and importance:**

Divergence insufficiency is common in the spinocerebellar ataxia variants and is thought to be secondary to atrophy of brainstem structures involved in the control of ocular vergence. Strabismus surgery in these patients may be complicated by limited response or even rapid regression despite augmented surgery as suggested for divergence insufficiency in the setting of neurologic disease. Patients should be counseled on these risks as well as the potential for multiple procedures in order to achieve surgical success.

## 1 Introduction

The spinocerebellar ataxias are a group of genetic disorders caused by autosomal dominant polyglutamine repeats in the *MJD* gene. Spinocerebellar ataxia type 3, or Machado-Joseph disease (MJD), is the most common subtype, and is characterized by progressive degeneration of the cerebellum and brainstem with onset in the third to fourth decade of life.^1^ Patients suffer from ataxia, dysphagia, and vestibular abnormalities; severe weakness and paralysis can occur in end-stage disease.

Strabismus is common in MJD, with 64–75% of patients endorsing diplopia.^1^^,^^2^ Patterns of strabismus in MJD are heterogeneous, particularly in younger patients, and include exotropia, hypotropia, and skew deviation. Mild abduction deficits are common and may be associated with either exo- or esodeviations.^2^ Commonly, however, ocular motility disorders in MJD are frequently a result of vergence abnormalities, typically producing secondary divergence insufficiency esotropia, or that in which the angle of esodeviation is greater at distance than at near.^3^ The etiology in these cases is thought to be due to a disruption in the central vergence pathway, differing from age-related distance esotropia (ARDE), which is attributable to degenerative anatomic changes in the orbital pulley system between the lateral and superior rectus muscles.^4^^,^^5^ ARDE has previously been shown to respond well to augmented medial rectus recessions with the target angle being twice the distance esotropia.^4^ However, this approach may not be as effective in secondary divergence insufficiency esotropia secondary to MJD.

We present a patient with divergence insufficiency esotropia secondary to MJD who exhibited complete regression of the preoperative deviation within one week after augmented bimedial rectus recessions. Following a second surgery with bilateral lateral rectus resections, orthotropia was achieved.

## 2 Case report

A 53-year-old female presented to the ophthalmology clinic with intermittent horizontal diplopia, worse at distance than at near. The diplopia began eight years prior and was associated with the onset of gait unsteadiness. While prism spectacles had been previously prescribed and provided interval resolution of diplopia, she now reported worsening of symptoms refractory to conservative management.

**Fig. 1:**
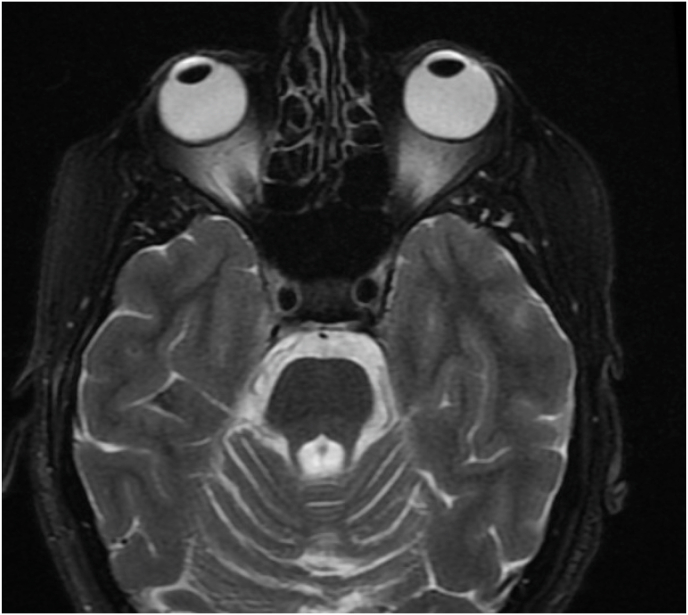
T2-weighted magnetic resonance image of displaying atrophy of the cerebellum and cerebellar peduncle bilaterally consistent with spinocerebellar degeneration in Machado-Joseph Disease.

A few years after diplopia onset, the patient was diagnosed with MJD via genetic testing. Neuroimaging demonstrated prominent atrophy of the cerebellum and brainstem (Fig. 1). She suffered from ataxia and lower extremity rigidity. While no family members had confirmatory genetic testing, the patient's mother and maternal grandfather had similar symptoms. Additional past medical history was notable for anxiety and depression. The patient underwent laser corrective surgery in both eyes in young adulthood for moderate myopia.

On examination, the patient had a best-corrected visual acuity of 20/20 in both eyes. Extraocular movements were full except for mild, −1 abduction deficits bilaterally. Alternate prism cover testing to a distance fixation target revealed an esotropia of 16 prism diopters (PD) in primary gaze which increased to 25 PD in right and left gaze. With a near fixation target, there was 1 PD of exophoria, and the patient demonstrated 100 arc seconds of near stereopsis by Randot testing. There was end-gaze nystagmus, becoming right beat on right gaze, left beat on left gaze, and slightly upbeating on upgaze. External exam demonstrated dermatochalasis of the upper eyelids bilaterally. The remainder of the slit lamp and dilated funduscopic examination was unremarkable.

Given the presence of largely full extraocular motility and superior sulcus abnormality, ARDE, or “sagging eye syndrome,” was considered.^5^ Preoperative magnetic resonance imaging (MRI) of the orbits revealed normal lateral and superior rectus anatomy and trajectory, however. Intraoperative forced duction testing were noted to be free in both eyes. The patient then underwent 4.5 mm bilateral medial rectus recessions on adjustable sutures, correcting for twice the measured angle of deviation as recommended by Demer et al. for the surgical management of divergence insufficiency esotropia.^4^ The patient was orthotropic and diplopia-free one hour post-operatively and no adjustment was required.

**Fig. 2:**
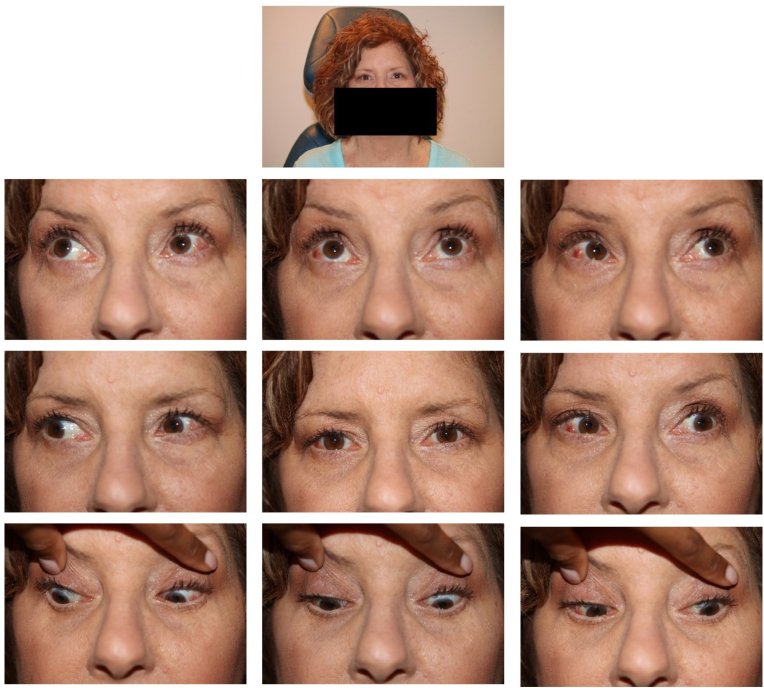
Extraocular movements two months after bilateral lateral rectus resection.

Four days after surgery, however, the patient's diplopia recurred. She was examined one week post-operatively and found to have regression of the esotropia to the full pre-operative level of 16 PD at distance and orthotropia at near. The patient was taken back to surgery six months later, during which forced duction testing was again noted to be free in both eyes and the medial rectus positions were confirmed to be 4.5mm recessed from their anatomic insertions. The lateral recti were examined and were noted to have a normal-appearing insertion, caliber and trajectory over the globe. As such, 2.5 mm bilateral lateral rectus resections on adjustable sutures were performed, correcting for slightly less than 15 prism diopters of esotropia. The patient was orthotropic post-operatively but demonstrated a small residual esophoria on post-operative day two. As it was felt that she had a tendency for esotropia regression, the lateral rectus resections were augmented to 3.5 mm bilaterally. Following this, she continued to remain orthotropic and diplopia-free by post-operative month two without developing a consecutive exodeviation (Fig. 2).

## 3 Discussion

We describe a case of divergence insufficiency in the setting of MJD that recurred completely within one week of augmented medial rectus recessions, an approach previously shown to be effective for the management of divergence-insufficiency esotropia, and required additional augmented lateral rectus resections to achieve orthotropia.

Divergence insufficiency in MJD has been postulated to be related to atrophy of the nucleus reticularis tegmenti pontis within the dorsal pons, an area shown to play a role in vergence and accommodation in animal studies.^6^ Histopathologic post-mortem examination of a patient with MJD demonstrated substantial degeneration of the pontine reticular formation while the oculomotor, abducens, and trochlear nerve pathways were relatively spared.^7^ Aside from strabismus, other common ocular abnormalities in MJD include slow saccades, gaze-evoked nystagmus, and micro-opsoclonus. Together, these ocular manifestations may impart significantly decreased vision-related quality of life for patients with spinocerebellar ataxia.

The presentation of esotropia worse at distance than at near should also prompt the consideration of ARDE or “sagging eye syndrome”, in which age-related degenerative changes of the orbital pulley system, as evidenced by an inferiorly displaced lateral rectus on MRI, produce abduction deficits and corresponding divergence insufficiency. “Heavy eye syndrome” is also an important differential diagnosis, classically in a myopic eye, and can be diagnosed based on clinical history and the appearance of superotemporal globe prolapse with lateral rectus inferior displacement and superior rectus medial displacement. The current patient's MRI demonstrated normal anatomy of the orbits and extraocular muscles, but it is possible that her divergence insufficiency additionally incorporated a component of ARDE that was too mild to be visualized on neuroimaging, especially given the patient's superior sulcus abnormalities.

Esotropia in MJD and other cerebellar diseases can be successfully treated with prisms, botulinum toxin injection, and strabismus surgery.^8^ Divergence insufficiency esotropia has been shown to be associated with a significantly lower surgical dose-response compared to non-divergence insufficiency esotropia, with each millimeter of medial rectus recession producing fewer prism diopters of correction.^9^ As such, augmented medial rectus recession has been recommended for divergence insufficiency, with several surgical tables proposed in the literature.^4^^,^^9^

Furthermore, divergence insufficiency esotropia secondary to degenerative neurologic disorders often requires even larger surgical treatments to achieve success: a recent review of five patients with spinocerebellar ataxia who underwent bilateral medial rectus recession for esotropia found that these patients had significantly greater under-correction one week post-operatively than patients without neurologic disease.^10^ In our patient, while augmented recessions produced an excellent result in the immediate post-operative period, there was, prominently, complete regression of preoperative deviation within one week of surgery. These findings highlight the challenges in the management of this condition and may assist in the counseling of patients regarding post-operative course and the potential requirement for further surgery to achieve success. Late adjustment several days after surgery may be considered. Lastly, given the prominent dysfunction of vergence, lateral rectus resections may be considered ahead of medial rectus recessions in these cases.

The present case highlights the special considerations in managing strabismus in patients with MJD and other degenerative disorders of the cerebellum and brainstem. The increased risk of under-correction and the potential need for subsequent surgery facilitates management of patient expectations and aids the surgeon in favoring slight over-correction. While diplopia is most common in SCA3, the application of these treatment paradigms for the other spinocerebellar ataxias may be an important area of future investigation.^11^ Notably, it is difficult to directly compare the management of cerebellar esotropia in which there are pathologic central vergence mechanisms to that of sagging or heavy eye syndrome in which anatomic changes disrupt abduction function. As such, it is important to delineate the underlying etiology when deciding on the ultimate management plan for these patients. While the ocular motility abnormalities in the spinocerebellar ataxias pose unique challenges, resolution of diplopia in many cases, as with our patient, is ultimately possible.

## Patient consent

Consent to publish the case was not obtained as this report does not contain personal information that could lead to identification of the patient.

## Funding

No funding or grant support

## Authorship

All authors attest that they meet the current ICMJE criteria for Authorship.

## Declaration of competing interest

The authors report no conflicts of interests.
